# Spastic Paralysis; Talipes Equino-Varus*A Clinical Lecture. Abstracted by A. M. Phelps, M.D., Professor of Orthopedic Surgery, Post-Graduate School and Hospital, and in the University of City of New York; Surgeon to the New York City Hospital.

**Published:** 1895-03

**Authors:** Edmon Owen

**Affiliations:** London, England, Surgeon to St. Mary’s Hospital


ATLANTA

### Medical and Surgical Journal.

VOL. XII. MARCH, 1895. No. 1.

LUTHER B. GRANDY, M.D., MANAGING EDITOR. MILLER B. HUTCHINS, M.D., BUSINESS MANAGER.

### *ORIGINAL COMMUNICATIONS.*

### SPASTIC PARALYSIS; TALIPES EQUINO-VARUS.*

BY MR. EDMON OWEN, LONDON, ENGLAND,

Surgeon to St. Mary's Hospital.

The next case I have to show is a very interesting one. T. G., eleven years of age, came into the hospital in May, 1893. He was then ten and a half years of age, and was the subject of spastic paraplegia—that is to say, the reflex action in his lower extremities was uncontrolled, because of some affection of the spinal cord. The cells of the anterior cornu of the gray crescent of the cord are in connection with two sets of filaments—motor and sensory. The gray crescent is, in fact, a small, independent brain, responsible to the supreme authority of the encephalon. If we cut off the connection between the gray matter and the encephalon, there can evidently be no longer any direct control of the gray nerve-tissue; thus, for instance, on gently pinching the leg, we get spasmodic and uncontrolled contraction of the muscles of the limb. The reflex action is ordinarily controlled by inhibitory filaments running

*A Clinical Lecture. Abstracted by A. M. Phelps, M.D., Professor of Orthopedic Surgery, Post-Graduate School and Hospital, and in the University of City of New York; Surgeon to the New York City Hospital.

from the brain to the gray matter of the cord through the antero-lateral column of the cord; and if anything happens to interfere with the integrity of these filaments, the reflex acts lose inhibition and run riot. They had run riot in this boy. As he attempted to walk, contact between his foot and the ground caused spasmodic contraction of the muscles to take place, and he walked in the manner characteristic of spastic paraplegia, as I will demonstrate shortly in another case. He walked with stiffened legs, scraping his toes along the ground. In this boy the spastic paraplegia was not extremely well marked, but it was sufficiently obvious. There was spasmodic contraction of the calf muscles particularly, causing elevation of the heels, so that as he walked his toes were constantly catching on the ground. Moreover, the feet were constantly extended and inverted, in the position of talipes equino-varus.

The question was, what could be done for him? Through some early disease of the antero-lateral columns of the cord he had lost inhibition in his leg and feet centers, and it was altogether a most unpromising case for treatment. But we thought we would give the boy a chance by the open operation of Phelps, of New York, for talipes equino-varus. The result is that he now stands with his feet perfectly flat; there is neither inversion nor eversion, and, although there is still some clasp-knife action, he walks, so far as my part of the business is concerned, a perfect plantigrade. You will see the high stepping action as he goes along the floor, but, fortunately, his central nervous affection has greatly improved.

The case has made a considerable impression upon me, because, from a surgical point of view, it was extremely unpromising. I can remember the time when a surgeon would have refused to operate upon a case of talipes equino-varus, or any other form of talipes, which was secondary to central nervous disease, because the outlook was so poor. All such miserable cripples were, therefore, left without efficient treatment, and were allowed to drift on from bad to worse. I would not have operated on this boy had I not been particularly conversant with the operation of Phelps—a man who has done a great deal for orthopedic surgery, and who is, by the by, a general surgeon, not a special orthopedist. I think the time is coming when all bad cases of talipes equino-varus, except in very young children, will be operated upon by this open method. It seems to me, at least, to be inevitable. Here, truly, is a happy result of the thorough operation. All the credit of it is due to the large views and bold treatment of my American colleague, Dr. A. M. Phelps. I am not depreciating specialism altogether, but I have no hesitation in saying openly that I think specialism is going a little too far. May I here remark that probably the greatest advance that has been made in recent years in connection with the treatment of skin diseases was made by a general, not a special, physician—the treatment, namely, of inveterate cases of psoriasis by thyroid extract. If a man works within too narrow limits he is apt, I think, to lose sight of great principles, and take a contracted view of his surroundings. I do not say that he *is*, but certainly he is apt to be, like a man working in a valley. And in his work he is apt to develop a certain amount of professional myopia.

### PHELPS'S OPERATION.

A word or two with regard to Phelps's operation:

The old-fashioned and orthodox treatment of club-foot consisted in the subcutaneous division of tendons and fascia—division of the tibialis posticus, the flexor digitorum, and, perhaps, the plantar fascia. Then, with a good deal of subsequent manipulation and tedious working with a mechanical Scarpa's shoe, the foot was got into a more or less satisfactory position. Afterwards the tendon of Achilles was divided. This large tendon, you remember, was divided last of all. It was left for the purpose of acting as a fixed point, so that from it the surgeon might be able to exert, with a Scarpa's shoe, a certain amount of flexion and eversion. But if you happen to be dealing with a slight case of talipes equino-varus, it will very likely suffice if you divide only the tendon of Achilles. When this is effected you may be able to correct a very considerable amount of inversion as well as extension of the foot. I would, therefore, strongly advise, in every case, division of that structure first. That is a great point, but not an original one, in Phelps's operation. It is characteristic of Phelps's operation that, instead of dividing the inverting structures subcutaneously, the open method is employed, so that the surgeon can see exactly what he is doing, and thus divide nothing that does not require division, and everything that does.

(The last paragraph does not quite state all. The other reason, and by far the most important, is that the skin, cellular tissue, and fibrous tissue on the inner side of the foot are short, and these tissues must be lengthened, either by cutting, tearing, or stretching, before the foot can be brought to a super-corrected position, and cutting is the least harmful and most rapid—hence the open cut. —PHELPS.)

The incision is made, as I show you in this other child, from the dorsum of the foot across the inner side, just over the head of the astragalus, and is carried down to the sole. The internal saphenous vein is possibly divided, though it is often seen and avoided. The deep fascia has then to be cut, as it covers the abductor hallucis; then the tendon of the tibialis posticus which supports the head of the astragalus, and the tendon of the flexor longus digitorum underlying the head of the astragalus. Going a little further, the surgeon opens a joint between the astragalus and scaphoid. Now comes what I consider to be the most important point in the whole operation—the anterior part of the internal lateral ligament is freely cut. You remember how this ligament is arranged. The anterior fibers are not connected with the astragalus, but run over it to be attached to the scaphoid bone. The anterior part of the internal lateral ligament is peculiarly tight and resistant in talipes equino-varus, and, more than any other structure, requires attention. As soon as that is done, the foot is everted and the joint between the astragalus and the scaphoid opened up. The other resisting structures in the foot are then dealt with. Amongst them will come, I dare say, the middle piece of the plantar fascia, which is the strongest part, and, very likely, the flexor brevis digitorum. Then the inferior calcaneo-scaphoid ligament has to be divided because it is holding the tuberosity of the scaphoid up against the sustentaculum tali. The position of the foot is to be improved by increasing the length of the inner border, and that can only be done by opening the joints between the astragalus and scaphoid, a measure which is impossible without division of the inferior calcaneo-scaphoid ligament. After every cut the surgeon wrenches the foot into a slightly improved position; he goes step by step, feeling his way, as it were, with the tip of his finger and the end of his scalpel. Perhaps before the foot can be got into the proper position the long and the short calcaneo-cuboid ligaments have to be divided. After that the surgeon gives another wrench and gets the foot into an over-corrected position. He dresses the wound lightly with some antiseptic gauze, loosely filling the large cavity, and then he secures the foot in lateral splints of house flannel and plaster of Paris.

It may not be amiss to compare, for a moment in passing, this operation with other radical operations on the foot which consisted in the removal of the wedge-shaped piece from the outer border of the foot. If the apex of the wedge is brought far enough inwards and the base is sufficiently wide, the foot can then be straightened out and brought flat. But this improvement is obtained at the expense of the length of the foot. Different varieties of these operative procedures bear the names of different surgeons—Davies Colley and Richard Davy; and there is yet another, and a very excellent one it is, which cousists in the removal of the astragalus: it bears the name of a well known provincial surgeon—Lund, of Manchester. These various procedures have emanated during the last few years from pioneers in orthopedic surgery, all of whom, by the by, were general surgeons.

All of these operations, useful as they have been in the evolutions of the surgery of club-foot, effected their improvement by shortening the external border, or sacrificing some part of the foot; but Phelps's operation improves the position of the foot—not by shortening or sacrificing anything, but by lengthening the internal border of the foot, and I am satisfied that it is of a very great importance.

The wound having been dressed in the case of this boy, operated on as described, on May 16th, the foot was wrenched around into the over-corrected position and encased in lateral splints of house flannel and plaster of Paris. Then for five weeks it was not interfered with. Only to-day the second dressing was taken off, two weeks having elapsed since the first was removed. When the dressing was removed the wound was almost healed, and, as you will see, it must have been an extensive one originally. Mr. Kellock, who, with me, operated on one of this boy's feet some time ago, suggested and carried out an ingenious modification in the detail: As soon as the foot is lengthened out there is a considerable amount of slack skin upon the dorsal and outer aspect of the foot; so, after the deep operation-wound on the inner side of the foot had begun to granulate, Mr. Kellock raised a large flap of this redundant integument and slipped it into the wound. This graft has done well, and its growth has materially expedited the healing.

(No matter how wide the wound has gaped, in my experience it has always filled in perfectly within six weeks, and within a short time the redundant skin on the outside of the foot has been absorbed. With these observations in mind, I think I would hardly resort to a plastic operation in any case, although I would not condemn the practice.—PHELPS.)

The old treatment by Scarpa's shoe required a great deal of attention on the part of the surgeon, who required, in private practice, to make almost daily visits to see how the case was going on, to assure himself that the foot was bearing the restraint, and to alter the screws. According to the new procedure, the foot is put up in plaster of Paris and so left for three or four weeks, the patient being allowed to walk about within a week of the operation.

(Mr. Owen is right in teaching that contraction following paralysis should be lengthened by operation. The senseless, prolonged, painful stretching treatment, followed by some orthopedists, is to be deplored. It will be abandoned in the near future. It is as unscientific to attempt, by machines, to stretch these contracted muscles and tendons, as it is to follow the same plan of treatment with the remunerative tendo-Achillis Dupuytren contraction, now so popular with certain mechanicians. These paralyzed muscles should be lengthened by interposing an abundance of new tissue, and not by stretching. The latter nearly always relapses, making it remunerative for the mechanic, while the cases operated upon do not, or, at least, very seldom, relapse, and the usefulness of the foot is very much superior to those treated by stretching.—PHELPS.)

